# Imbalanced nutrition and increased dietary inflammatory index in children with autism spectrum disorder: associations with neurodevelopmental disorders

**DOI:** 10.3389/fnut.2025.1705682

**Published:** 2025-11-19

**Authors:** Jing Shen, Junqiao You, Wenjing Wang, Wenli Yang, Wenli Zhao, Jianan Zhao, Huini Ding, Yuandi Xi, Hongmei Huang

**Affiliations:** 1 School of Public Health, Capital Medical University, Beijing, China; 2 Department of Clinical Nutrition, Beijing Children's Hospital Affiliated to Capital Medical University, National Children's Medical Center, Beijing, China; 3 School of Public Health, Capital Medical University, Beijing, China

**Keywords:** nutrition, neurodevelopment, autism spectrum disorder, dietary patterns, inflammation

## Abstract

**Background:**

The incidence rate of autism spectrum disorders (ASDs) has been rising; this study aimed to explore the relationship between nutritional intake and neurodevelopment in children with ASDs.

**Methods:**

This study is a case–control cross-sectional comparison including 50 children with ASDs and 50 typically developing controls. A total of 500 children with neurodevelopmental disorders and 500 typically developing (TD) children were recruited based on clinical diagnosis to compare levels of inflammation. Among them, 50 children were diagnosed with ASDs based on DSM-5 criteria by experienced developmental behavioral pediatricians. A total of 50 typically developing children were matched with the 50 children with ASDs. Dietary surveys, Autism Diagnostic Observation Schedule (ADOS), and physical development evaluations were conducted on these 100 children.

**Results:**

The children’s dietary inflammatory index (C-DII), systemic immune inflammatory index (SII), and systemic inflammatory response index (SIRI) in the ASD group were significantly higher compared to the TD group (*p*<0.05). A positive correlation was observed between C-DII and SII (*r* = 0.323, *p* = 0.022), as well as SIRI (*r* = 0.283, *p* = 0.046). The dietary diversity in the ASD group was lower than that of the TD group, with a higher prevalence of picky eating behaviors in the ASD group. After adjusting for demographic characteristics, the C-DII may be correlated with ASD [odds ratio (OR) = 1.842, 95% confidence interval (CI) = 1.114 ~ 3.046, *p* = 0.017].

**Conclusion:**

Children with autism spectrum disorders (ASD) displayed higher C-DII scores. The results of this study suggest that there may be a correlation between C-DII and the neurodevelopment of children with ASD, not causality. Inflammatory dietary patterns may represent a modifiable factor linked to neurodevelopmental outcomes.

## 1 Introduction

Autism spectrum disorders, commonly referred to as autism, manifest in early childhood and are characterized by significant impairments in social communication and the presence of repetitive behaviors, which profoundly impact children’s social functioning and overall quality of life. In recent years, the incidence of ASDs has been on the rise, emerging as a global concern, with an estimated prevalence of approximately 7‰ among children in China (1). Notably, over 70% of children diagnosed with ASDs encounter nutritional challenges (2).

Current research suggests that the factors affecting children’s neurological development mainly include both genetic and environmental factors. Research indicates that the nutrients ingested can influence the development of the central nervous system and play an essential role in neurodevelopment (3). Nutritional factors, as modifiable lifestyle elements, suggest that feeding methods and the intake of various foods and nutrients may be associated with the neurodevelopment of children. A meta-analysis regarding the dose–response relationship between the duration of breastfeeding and the risk of ASDs has shown a significant association; as the duration of breastfeeding increases, the risk of developing ASDs decreases (4). A review focusing on micronutrients and brain development has elucidated that children who receive adequate nutrition are more likely to reach their developmental potential in cognitive, motor, and socioemotional domains, resulting in positive societal outcomes (5). It is arguable that overall dietary patterns, rather than single nutrients, exert a more comprehensive impact (6). Exploring a comprehensive dietary index can more accurately reflect the association between dietary quality and the neurodevelopment of children.

Furthermore, inflammation is increasingly acknowledged as a significant factor in central nervous system impairment in both developing children and adults (7). Inflammation is a ubiquitous underlying mechanism linking diet and cognitive functioning (8). The dietary inflammatory index (DII) has been established in nutritional research to characterize and quantify the overall inflammatory potential of dietary patterns (9). Current studies have observed that high DII scores were associated with lower overall cognitive function and a higher risk of mild cognitive impairment (MCI) (10–15). Evidence indicated that high DII scores and pro-inflammatory diets in children were closely related to common pediatric conditions that impact central nervous system functionality, such as obstructive sleep apnea–hypopnea syndrome (OSAHS) (16). The higher the children’s dietary inflammatory index (C-DII), the greater the impact on the severity of children’s OSAHS (16).

The systemic immune-inflammatory index (SII) and systemic inflammatory response index (SIRI) are emerging biomarkers that reflect chronic systemic inflammation. The SII highlights platelet-associated immune mechanisms, while the SIRI comprises monocyte counts, thereby improving its capacity to reflect immune regulatory processes (17). This offers a more comprehensive reflection of the potential systemic inflammatory status and immune responses pertinent to neurodevelopment than a single biomarker could achieve. Studies have shown that children with obesity, particularly those with metabolic syndrome, exhibit higher SII levels (18). Investigations have demonstrated that behaviors such as nutritional approaches and physical activity may modulate immune function, thereby impacting neurological development and systemic health (19, 20). These findings emphasize the impact of multiple factors, including nutritional intake, other behavioral patterns, and immune response, on neurodevelopmental outcomes. Diet-related inflammation may affect brain development through pathways such as cytokine activation, oxidative stress, alterations in gut microbiota, or changes in blood–brain barrier integrity (21). The comorbidities, sensory preferences, and feeding selectivity of ASDs also may have an impact on nutrition and inflammation (22). Therefore, it is necessary to explore the relationship between nutrition, inflammation, and ASDs.

Currently, there is a paucity of research on the relationship between C-DII and ASDs, and the association between C-DII and ASDs remains to be clarified. Is a higher C-DII associated with an increased risk of ASDs independent of confounding variables? The objective of this study was to explore whether there is a correlation between nutritional status and neurodevelopment of children with ASD. Investigating this area is crucial for improving neurodevelopmental outcomes through dietary interventions.

## 2 Methods

### 2.1 Study population

Previous studies have found that inflammatory markers may be elevated in individuals with neurodevelopmental disorders. We recruited 500 children with neurodevelopmental disorders and 500 typically developing (TD) children (aged 3.0 ± 1.70 years) from Beijing Children’s Hospital, affiliated with Capital Medical University, and a community in the Fengtai District of Beijing, to compare their levels of inflammation. All participants were enrolled based on their clinical diagnosis; the 500 TD children were selected from neurodevelopmentally typical children in outpatient and community settings. They received a clinical diagnosis and did not have ASDs or other neurodevelopmental disorders. By adjusting for covariates, including sex and age, in the logistic regression model, we aimed to observe the synergistic effect of SII and SIRI on neurodevelopment.

Within the 500 children with neurodevelopmental disorders, 50 children were diagnosed with ASD through professional clinical neurodevelopmental assessments, with ages ranging from 1 to 3 years. We included these 50 children with ASD in the ASD group. From the 500 TD children, 50 TD were selected to be matched with the ASD group in a 1:1 ratio, as the TD group, based on sex and age. At the same time, dietary surveys, behavioral observation and evaluation, physical development measurements, and birth status such as delivery gestational week and history of birth asphyxia were conducted on these 100 research subjects. Before enrollment, the legal guardians of all eligible participants confirmed and provided informed consent. Children with other independent neurodevelopmental disorders, mental illnesses, or other health conditions were excluded from this study.

### 2.2 Dietary survey and assessment

The feeding behaviors and dietary surveys for children were designed with reference to other similar studies and the “Dietary Guidelines for Chinese Residents” (23). We utilized the standardized food frequency questionnaire (FFQ) to collect comprehensive dietary data. Trained interviewers conducted face-to-face surveys with the guardians of participants in community settings or outpatient clinics to collect dietary intake information when the children were 1.5 years old. The daily nutrient intake for children was calculated using the Chinese Food Composition Table. At the same time, the estimated average requirements (EER/EAR) were based on the Dietary Reference Intakes for Chinese (DRIs). The EAR represents the average nutrient requirement for individuals within specific sex, age, and physiological condition categories.

### 2.3 Calculation of DDS and C-DII

The dietary diversity scores (DDS) were calculated according to the Food and Agricultural Organization of the United Nations (FAO) guidelines titled “Guidelines for Measuring Household and Individual Dietary Diversity,” categorizing daily food intake into nine groups (24): (1) cereals, potatoes and mixed beans; (2) vegetables; (3) fruits; (4) meat and poultry; (5) eggs; (6) milk and dairy products; (7) soybeans and nuts; (8) aquatic product; (9) oil. A child must consume at least 10 g of a food from one distinct food group within the 24-h dietary record to earn one point, with a maximum score of 9. The scoring ranges from 1 to 9, where a score above 6 indicates no risk of nutrient deficiency. This information was collected through parental recollection of the food types consumed by their children in the past 24 h.

The dietary inflammatory index was used to assess the inflammatory potential of diet, according to Shivappa et al. (25). 25 types of food parameters were used to calculate the C-DII scores (26). Based on the mean daily global intake of 25 dietary components, $x_{\text{global}}$ and $S_{\text{global}}$ , from which the calibrated value (*Z*) of the average daily intake of a dietary component (*x*) per person was calculated as $Z=x-x_{\text{global}}/S_{\text{global}}$ . The *Z*-score was converted to a percentile and multiplied by 2 minus 1 to obtain a symmetric distribution centered at 0, ranging from −1 to +1, which is denoted as *p*. The effect of the 25 dietary components on the inflammation-related indices was combined and converted into an “inflammatory effect score (denoted as *e*),” which has a lower limit of −1 and an upper limit of 1, with closer to −1 representing higher anti-inflammatory capacity and closer to 1 representing higher pro-inflammatory capacity. $\mathrm{DII}=\sum_{i=1}^{n}P_{i}\times e_{i}$ , where *n* is the number of dietary components included.

### 2.4 Neurodevelopmental assessment

All participants were consulted by at least one experienced developmental behavior pediatrician, according to DSM-5 diagnostic criteria (27). Neurodevelopmental scores were assessed using the Children’s Neuropsychological Development Scales and the Autism Diagnostic Observation Schedule (ADOS). The Children’s Neuropsychological Development Scales comprise five domains: gross motor skills, fine motor skills, adaptive behavior, speech, and social behavior. Intellectual development is represented by the Development Quotient (DQ), where DQ = Average Score of the five domains/actual chronological age×100. The ADOS includes social communication and repetitive behaviors.

### 2.5 Physical development measurement

Physical measurement indicators include weight, height, and body mass index (BMI). Weight in kilograms and height in centimeters were measured using calibrated electronic scales and height meters, ensuring the accuracy of both instruments before use and retaining results to one decimal place. BMI was calculated as weight (kg)/[height (m)]^2^. The World Health Organization (WHO) Anthro Plus and WHO Anthro software were employed to calculate the Height for Age *Z*-score (HAZ), Weight for Age *Z*-score (WAZ), and BMI for Age *Z*-score (BAZ). Based on BAZ, children’s nutritional status was categorized into severe obesity, obesity, overweight, normal, emaciated, and severely emaciated.

### 2.6 Laboratory measurements

Blood samples were collected from individuals who had fasted since 8 p.m. the previous night. The absolute counts of neutrophils, lymphocytes, monocytes, and platelets in peripheral blood were analyzed using an automatic analyzer. SII and SIRI were calculated based on the absolute platelet count in peripheral blood (P; ×10^9^/L), the neutrophil count (N; ×10^9^/L), the monocyte count (M; ×10^9^/L), and the lymphocyte count (L; ×10^9^/L), using the formulas SII=P × N/L and SIRI=N × M/L (28).

### 2.7 Statistical analysis

The statistical description and inference of the data were based on its characteristics, with suitable descriptive indices and hypothesis testing methods selected. Continuous variables were expressed as medians (interquartile ranges, IQR) when they were non-normally distributed; otherwise, a mean ± standard deviation (SD) was used in the normal distribution. For comparisons of non-normally distributed quantitative data between two groups, the Mann–Whitney U test was employed. Categorical data were expressed as counts and percentages (%), and comparisons between two groups of categorical data were performed using the chi-square test. Comparisons between groups were made using grouped *t*-tests, analysis of variance (ANOVA), or Wilcoxon rank-sum tests, depending on the distribution of the data. Multiple linear regression models were adjusted by confounding factors to assess the relationship between C-DII and the core symptoms of ASDs and neurodevelopmental domains. All *p*-values were False Discovery Rate (FDR) corrected using the Benjamini–Hochberg program to control false positive detection rates. All regression models have undergone hypothesis testing. No severe multicollinearity has been confirmed through variance inflation factor testing; the linear hypothesis between continuous independent variables and outcomes has been confirmed through component residual plots and polynomial term testing. A multivariate binary logistic regression model was used to evaluate the association of C-DII with ASDs. Statistical significance was set at a two-sided *p* < 0.05. Statistical Package for Social Sciences (SPSS) Statistics 26 (IBM Corporation, Armonk, NY, USA) was used to perform statistical analyses.

## 3 Results

### 3.1 Demographic characteristics and blood inflammation index of participants

The comparison of characteristics between 500 children with neurodevelopmental disorders and 500 TD children is shown in Table 1. Among the 1,000 participants, 63.4% were male. The SII and SIRI of children with neurodevelopmental disorders were higher than those of the TD children (*p* < 0.001). Table 2 shows the synergistic effect of SII and SIRI on neurodevelopment using a binary logistic regression model.

**Table 1:** Demographic characteristics with neurodevelopmental disorders and TD children.

Independent variables	Total (*N* = 1,000)	Neurodevelopmental disorders (*N* = 500)	TD (*N* = 500)	*p*
Age (years old)	3.77 (2.34, 6.30)	4.14 (2.47, 7.01)	3.60 (2.22, 5.09)	0.002**
Sex				<0.001**
Male	634 (63.4)	353 (70.6)	281 (56.2)	
Female	366 (36.6)	147 (29.4)	219 (43.8)	
Inflammatory index				
SII	166.43 (103.62, 281.70)	216.05 (120.20, 393.42)	140.94 (91.95, 209.23)	<0.001**
SIRI	0.22 (0.13, 0.40)	0.31 (0.16, 0.69)	0.18 (0.11, 0.29)	<0.001**

Categorical and continuous variables were shown as *n* (%) and median (P25, P75). SII, systemic immune inflammatory index; SIRI, systemic inflammatory response index.**p < 0.01.

**Table 2:** Synergistic effect of SII and SIRI on neurodevelopment by logistic regression model.

Independent variables	Model1	*p*
	OR (95% CI)	
SII ^Model 1^	1.006 (1.004, 1.007)	<0.001**
SIRI ^Model 1^	1.566 (1.402, 1.731)	<0.001**

SII, systemic immune inflammatory index; SIRI, systemic inflammatory response index. Model 1, adjusted for age and sex; **p < 0.01.

### 3.2 Demographic characteristics, C-DII, SII, and SIRI of ASDs and TD

The general conditions, C-DII, SII, and SIRI of 50 children with ASDs and 50 TD children were compared in Table 3 and Supplementary Table S1. Of these participants, 70% were male, and there were no significant differences between the two groups regarding sex, age, or introduction of complementary feeding, HAZ, WAZ, or BAZ. However, there were differences in feeding patterns between the two groups (*p* < 0.05). The interquartile range (IQR) of the C-DII score was 2.89 (2.35, 3.21), with a range from −0.710 to 4.063. The SIRI was 0.13 (0.12, 0.37), with a range of 0.1 to 6.89. The SII was 132.13 (104.93, 262.71), with a range of 54.63 to 1411.36. The C-DII (*p* = 0.031), SIRI (*p* < 0.001), and SII (*p* < 0.001) of the ASD group were significantly higher than those of the TD group.

**Table 3:** Demographic characteristics and nutritional status of ASD and TD children.

Independent variables	Total	ASD (*N* = 50)	TD (*N* = 50)	*p*
Sex				0.081
Male	70 (70.0)	39 (78.0)	31 (62.0)	
Female	30 (30.0)	11 (22.0)	19 (38.0)	
Inflammatory index				
C-DII	2.89 (2.35, 3.21)	2.95 (2.74, 3.17)	2.61 (1.55, 3.32)	0.031*
SII	132.13 (104.93, 262.71)	262.22 (150.46, 430.19)	105.91 (87.93, 120.58)	<0.001**
SIRI	0.13 (0.12, 0.37)	0.36 (0.19, 0.87)	0.12 (0.11, 0.13)	<0.001**
DDS				<0.001**
1 ~ 3	5 (5.0)	5 (10.0)	0 (0)	
4 ~ 6	50 (50.0)	42 (84.0)	8 (16.0)	
7 ~ 9	45 (45.0)	3 (6.0)	42 (84.0)	
Be picky about food				<0.001**
Yes	46 (46.0)	34 (68.0)	12 (24.0)	
No	54 (54.0)	16 (32.0)	38 (76.0)	

Categorical and continuous variables were shown as *n* (%) and median (p_25_, p_75_). C-DII, Children’s dietary inflammatory index; SII, systemic immune inflammatory index; SIRI, systemic inflammatory response index. DDS, dietary diversity scores, not enough: 1 ~ 3; moderate: 4 ~ 6; adequate: 7 ~ 9. *p < 0.05, **p < 0.01.

### 3.3 Correlation between SII, SIRI, and C-DII

Using Pearson correlation analysis in 50 children with ASDs, this study demonstrated a positive correlation between C-DII and SII (r = 0.323, *p* = 0.022), as well as a positive correlation between C-DII and SIRI (r = 0.283, *p* = 0.046) (Table 4). There was no correlation between SII/SIRI and DDS, feeding patterns, and the timing of complementary food addition.

**Table 4:** The correlation between SII, SIRI, and C-DII.

Independent variables	SII		SIRI	
	*r*	*p*	*r*	*p*
C-DII	0.323	0.022*	0.283	0.046*
DDS	0.057	0.692	−0.003	0.981
Feeding patterns	−0.036	0.802	0.147	0.307
Add complementary food age	0.123	0.394	0.016	0.915

C-DII, Children’s dietary inflammatory index; SII, systemic immune-inflammatory index; SIRI, systemic inflammatory response index. DDS, dietary diversity scores. r, correlation coefficient. *p < 0.05.

### 3.4 Comparison of dietary situation in the ASD and TD groups

The dietary diversity of the ASD group was lower than that of the TD group (*p* < 0.001), and the phenomenon of picky eating in the ASD group was significantly higher than in the TD group (*p* < 0.001). In the ASD group, 68% of the children were picky eaters, compared to 24% in the TD group. There was no difference in appetite loss or poor feeding interaction between the two groups (Table 3).

### 3.5 Comparison of dietary energy and nutrient intake between ASD and TD groups

There were statistically significant differences in the intake of fat, total fatty acids, calcium, phosphorus, sodium, zinc, total vitamin A, vitamin C, vitamin D, thiamine, riboflavin, vitamin B_12_, and carotene among the ASD group. The intake of fat, total fatty acids, and sodium in the ASD group was higher than that in the TD group. In comparison, the intake of calcium, phosphorus, zinc, total vitamin A, vitamin C, vitamin D, thiamine, riboflavin, and vitamin B_12_ was significantly lower than in the TD group (Table 5). Table 5 shows the nutrients with statistically significant differences in intake between the two groups.

**Table 5:** Dietary energy and nutrient intake between ASDs and TD.

Nutrients	ASD (*N* = 50)	TD (*N* = 50)	*p*
Fat (g/day)	43.5 (33.75, 49.98)	31.18 (25.3, 41.47)	<0.001**
Total fatty acids (mg/day)	27.81 (21.81, 37.12)	15.15 (10.2, 21.67)	<0.001**
MUFA (mg/day)	13.82 (10.65, 16.44)	9.67 (7.73, 13.79)	<0.001**
PUFA (mg/day)	8.26 (6.22, 10.95)	3.98 (2.37, 5.56)	<0.001**
Ca (mg/day)	400.87 (231.27, 529.29)	661.4 (473.09, 856.46)	<0.001**
P (mg/day)	550.66 (374.45, 668.87)	679.74 (545.65, 937.78)	0.001**
Na (mg/day)	1, 738.84 (1, 381.61, 2380.12)	499.82 (375.05, 776.19)	<0.001**
Zn (mg/day)	5.25 (3.96, 6.22)	7.06 (5.32, 9.45)	<0.001**
Vitamin A (μgRAE/day)	404.44 (253.56, 501.58)	521.87 (399.36, 681.05)	<0.001**
Carotene (μg/day)	1, 497.13 (1041.84, 2163.66)	1, 164.67 (755.46, 1832.63)	0.038*
Vitamin D (μg/day)	7.23 (6.23, 8.97)	14.90 (10.83, 16.72)	<0.001**
Vitamin E (mg/day)	16.97 (13.06, 19.84)	12.1 (9.27, 16.38)	<0.001**
Thiamine (mg/day)	0.49 (0.39, 0.57)	0.68 (0.48, 0.86)	0.001**
Riboflavin (mg/day)	0.68 (0.42, 0.82)	0.87 (0.75, 1.22)	<0.001**
Vitamin B_12_ (μg/day)	2.15 (1.57, 2.93)	2.65 (2.05, 3.39)	0.022*
Vitamin C (mg/day)	45.75 (30.7, 57.7)	53.62 (35.57, 70.86)	0.034*

MUFA, monounsaturated fatty acids; PUFA, polyunsaturated fatty acids; Ca, calcium; P, phosphorus; Na, sodium; Zn, zinc; RAE, retinol activity equivalent; *p < 0.05, **p < 0.01.

### 3.6 Comparison of nutrient intake with EAR between ASD and TD groups

The average daily intake of carbohydrates, vitamin B_1_, vitamin B_2_, vitamin C, vitamin D, vitamin B_12_, and calcium per person in the ASD group was lower than that of the TD group. The proportion of boys in the ASD group whose protein intake reached EAR was higher than that of the TD group, while the proportion of girls in the ASD group whose protein intake reached EAR was lower than that of the TD group. The proportion of boys in the ASD group who reached the EAR for vitamin A intake was lower than that of the TD group, and the proportion of girls in the ASD group who reached the EAR for vitamin A intake was also lower than that of the TD group. The proportion of the ASD group with phosphorus intake reaching EAR was higher than that of the TD group. The proportion of zinc intake reaching EAR in the ASD group was roughly equal to that of the TD group (Supplementary Table S2).

### 3.7 Correlation between C-DII and neurodevelopmental domains in ASDs

The correlation between C-DII and various neurodevelopmental attribute scores was explored through multiple linear regression (Table 6). After adjusting for age, sex, feeding patterns, gestational week at delivery, history of birth asphyxia, and BAZ, we did not find a significant correlation between C-DII and the scores in the five domains of the Children’s Neuropsychological Development Scales, as well as the ADOS scores.

**Table 6:** The correlation between C-DII and neurodevelopmental domains.

Neurodevelopmental domains	C-DII	Age	Sex	Feeding patterns	Mode of delivery	History of birth asphyxia	BAZ
DQ							
β	−0.093	0.151	0.088	−0.017	0.290	0.269	0.124
*p*	0.530	0.312	0.542	0.906	0.049	0.062	0.398
Gross motor							
β	0.034	0.218	0.267	0.023	−0.025	0.128	0.005
*p*	0.830	0.177	0.090	0.885	0.874	0.404	0.975
Fine motor							
β	−0.253	0.065	0.078	0.085	0.239	0.124	0.241
*p*	0.094	0.664	0.589	0.566	0.105	0.388	0.109
Adaptability							
β	−0.288	0.075	0.210	0.006	0.171	0.222	0.157
*p*	0.056	0.614	0.148	0.969	0.238	0.122	0.287
Language							
β	0.285	0.095	−0.116	−0.070	0.397	0.250	0.031
*p*	0.051	0.511	0.407	0.622	0.006	0.074	0.829
Social behavior							
β	−0.179	0.148	−0.066	−0.100	0.246	0.270	0.022
*p*	0.236	0.329	0.649	0.505	0.097	0.065	0.884
ADOS (Social communication)							
β	−0.263	0.104	0.124	0.016	−0.294	0.175	0.066
*p*	0.096	0.505	0.414	0.918	0.058	0.245	0.671
ADOS (Repetitive behavior)							
β	0.156	0.205	−0.018	0.266	−0.302	−0.100	−0.030
*p*	0.288	0.166	0.900	0.072	0.039	0.475	0.839

BMI, Body mass index; BAZ, BMI for Age Z-score; C-DII, Children’s dietary inflammatory index. The model was adjusted for age, sex, feeding patterns, mode of delivery, history of birth asphyxia, and BAZ.

### 3.8 Correlation between C-DII and ASD

A generalized linear model, specifically a binary logistic regression model, was used to explore the correlation between C-DII and neurodevelopment (Table 7). After adjusting for age, sex, feeding patterns, picky eating, premature birth, history of birth asphyxia, and BAZ, the C-DII may be correlated with ASD [odds ratio (OR) = 1.842, 95% confidence interval (CI) = 1.114 ~ 3.046, *p* = 0.017].

**Table 7:** The correlation between C-DII and ASDs.

Model 1 Independent variables	OR (95%CI)	*p*
C-DII	1.842 (1.114, 3.046)	0.017*
Sex		
Male	Ref.	
Female	0.336 (0.123, 0.919)	0.034
Feeding patterns		
Exclusive breastfeeding	Reference	0.053
Artificial feeding	3.121 (0.631, 15.442)	0.163
Mixed feeding	3.152 (1.199, 8.286)	0.020
Delivery gestational week		
Full term	Reference	
Premature birth	—	0.999
History of birth asphyxia		
No	Reference	
Yes	—	0.999
BAZ	0.777 (0.502, 1.203)	0.258

BMI, Body mass index; BAZ, BMI for Age Z-score; C-DII, Children’s dietary inflammatory index. The model was adjusted for age, sex, feeding method, premature birth, history of birth asphyxia, and BAZ; *p < 0.05.

## 4 Discussion

NDDs represent a group of chronic developmental brain function disorders with a high prevalence, typically emerging during early developmental stages, often in childhood (29). The symptoms and functional impairments associated with NDDs may persist into adolescence and adulthood, underscoring the necessity for a life-cycle approach to diagnosis and management (30). NDDs exhibit a higher prevalence in boys, with a male-to-female ratio of approximately 4:1 among individuals with ASDs in this study, aligning with findings from another study (31). We found significant differences in the blood inflammation index between children with neurodevelopmental disorders and their typically developing peers across a sample of 1,000. Considering the importance of the inflammation index for cognitive development (10–15), we designed a matched case–control cross-sectional comparison with a 1:1 ratio. By analyzing whether there were differences in C-DII between children with ASDs and TD children, as well as the correlation between C-DII and blood inflammation index, we aimed to explore the relationship between nutritional intake, dietary inflammatory potential, and neurodevelopmental status in children with ASD, and to provide novel insights and methodologies for the identification of early intervention strategies.

C-DII has emerged as an objective indicator for assessing the inflammatory potential in children, facilitating the development of personalized intervention strategies aligned with individual dietary objectives to mitigate the risk of inflammation and associated health complications. This study indicated that the ASD group had higher C-DII, SII, and SIRI values. Additionally, C-DII was positively correlated with SII and SIRI. An increase in C-DII or adherence to a pro-inflammatory dietary pattern may be associated with poorer neurodevelopmental outcomes. This study suggested a possible link between a pro-inflammatory diet and neurodevelopmental features, not causality. This investigation’s results on food diversity in children with autism indicated that their food diversity was significantly lower than that of the TD group; 68% of children with ASDs were picky eaters. DDS serves as a reliable metric for assessing micronutrient deficiencies in children (32). Children with a higher DDS are generally associated with increased micronutrient intake (33). The ASD group exhibited significantly lower intakes of calcium, phosphorus, zinc, total vitamin A, vitamin D, thiamine, riboflavin, vitamin B_12_, and vitamin C compared to the TD group. The proportion of average daily nutrient intake meeting the EAR for calcium, vitamin D, thiamine, riboflavin, vitamin B_12_, and vitamin C was lower in the ASD group than in the TD group; 50% of children in the ASD group failed to meet the average calcium requirement, and only 30% achieved the average vitamin D requirement. This phenomenon may be linked to the food choices of children with ASDs, influenced by factors such as food type, texture, and appearance, leading to low food diversity or nutrient deficiencies.

Evidence suggests that C-DII may act as a mediator for the influence of physiological inflammatory biomarkers on cognitive development, particularly through its anti-inflammatory components. Additionally, our previous research has demonstrated a positive correlation between DII and blood inflammation (10, 11). A high consumption of pro-inflammatory foods such as sweets, desserts, and chips has been linked to increased systemic inflammation and cognitive decline (34). Previous investigations into DII have predominantly focused on adult populations (35–39), yielding inconsistent results. This study focused on Chinese children, providing reference points for the prevention and prognosis of ASDs in Chinese children. Children with ASDs exhibited similar dietary preferences, demonstrating a strong preference for carbohydrates, snacks, and processed foods such as sweets and fried foods, while rejecting green leafy vegetables. This pattern aligns with findings from other studies (40–44), which report that severe nutrient deficiencies in children with ASDs may result from selective eating behaviors, resistance to specific tastes or colors, feeding behavior problems (e.g., distractibility during meals and refusal to eat), and dietary habits influenced by varying degrees of gastrointestinal symptoms. The comparison of nutrient intake between the two groups was consistent with findings from previous studies (45–50). Studies have shown that deficiency in certain micronutrients, such as zinc and vitamin B_12_, may lead to neurodevelopmental disorders (51). We did not observe significant deficiencies in the three macronutrients or severe growth and developmental issues among children with ASDs. This may be attributed to their preference for staple foods, sweets, and fried foods, which ensured adequate intake of macronutrients, particularly excess fat. Nevertheless, deficiencies in micronutrients and essential minerals warrant attention. Early screening and intervention may help reduce ASD-related malnutrition and improve the dietary quality in these ASD patients.

Mechanistically, a pro-inflammatory diet may serve as a stimulus that elevates inflammatory markers and augments the inflammatory response, potentially leading to brain dysfunction and the development of neurodegenerative disease (52). Evidence indicates that increased oxidative stress and altered apoptosis contribute to the pathogenesis of neurodegenerative diseases (53). Free radicals are involved in the progression of cognitive deficits by interrupting synaptic transmission, impairing mitochondrial function, promoting neuroinflammation, and interfering with axonal transport, all of which can lead to neuronal loss in neurodegenerative diseases (54). Furthermore, dietary patterns can regulate intestinal immunity through gut microbiota. Adherence to a pro-inflammatory diet may disrupt the balance of gut microbiota, thereby affecting the gut-brain axis, contributing to immune dysregulation, and influencing neural development (55, 56). Anti-inflammatory diets may serve as non-pharmacological interventions to mitigate immune-related dysfunction in individuals with ASD (57, 58). Modulating dietary inflammation could complement pharmacologic or behavioral interventions. Inflammation and nutrition jointly influence both metabolic and neurodevelopmental pathways, suggesting a shared etiological risk factor underlying metabolic and neuropsychiatric disorders (59). This study targets metabolic and inflammatory pathways, highlighting the convergence between nutritional and molecular strategies for early intervention.

This study has several limitations. First, the cross-sectional design precludes any causal inference, and the possibility of reverse causality cannot be ruled out; that is, ASD-related behavioral patterns, such as picky eating and restricted food preferences, may themselves lead to pro-inflammatory dietary profiles. Second, the absence of validated dietary assessment tools means that the FFQ and 24-h recall methods relied largely on parental recall, introducing potential recall and reporting biases—for example, parents may forget certain snacks or inaccurately estimate weights and portions of food their children consumed. In this study, an artificial food mold, a booklet containing food quality estimation pictures, and a placemat marked with grids were used to facilitate portion estimation (60). Multiple imputation was used to address missing data, generating several complete imputation datasets through statistical modeling. Third, this study did not include socioeconomic and clinical variables. However, all participants were recruited from the same geographical region and city, which, to some extent, reduces the possibility of significant socioeconomic disparities caused by differences due to residence. Nonetheless, systematically collecting socioeconomic data in future research will be crucial. Fourth, a small sample size limits statistical power; the negative results we observed should be interpreted cautiously, as they may exist type II errors. These findings necessitate validation through subsequent large-sample, multi-center, prospective studies that further explore the underlying mechanisms. This study may help provide a basis for exploring early intervention approaches, such as lifestyle and dietary programs, aimed at improving ASD outcomes through dietary and inflammatory pathways, and may inform similar approaches in pediatric ASD research (61).

## 5 Conclusion

Children with ASDs showed higher dietary inflammatory scores and lower dietary diversity, suggesting a possible link between a pro-inflammatory diet and neurodevelopmental features. The C-DII may serve as a modifiable correlate of neurodevelopmental differences in ASDs. Future research should focus on longitudinal or interventional studies to determine whether dietary changes affect inflammation and neurodevelopmental outcomes. These findings highlight the potential clinical relevance of monitoring diet and inflammation status in children with ASDs.

## Data Availability

The original contributions presented in the study are included in the article/Supplementary material, further inquiries can be directed to the corresponding authors.
