# Supplementary material for: Imbalanced nutrition and increased dietary inflammatory index in children with autism spectrum disorder: associations with neurodevelopmental disorders
Source: Front Nutr. 2025 Nov 19;12:1705682. doi: 10.3389/fnut.2025.1705682 (PMC12672334; doi:10.3389/fnut.2025.1705682)
Supplement: Supplementary file 1 [file Table_1.docx]

**Supplementary Table 1.** Demographic characteristics and nutritional status of ASD and TD children.

|  | Total | ASD（N=50） | TD（N=50） | *p* |
| --- | --- | --- | --- | --- |
| Feeding patterns |  |  |  | 0.026* |
| Exclusive breastfeeding | 43（43.0） | 15（30.0） | 28（56.0） |  |
| Artificial feeding | 13（13.0） | 9（18.0） | 4（8.0） |  |
| Mixed Feeding | 44（44.0） | 26（52.0） | 18（36.0） |  |
| Add complementary food age | |  |  | 0.447 |
| ＜6 months | 9（9.0） | 4（8.0） | 5（10.0） |  |
| 6 months | 57（57.0） | 26（52.0） | 31（62.0） |  |
| ＞6 months | 34（34.0） | 20（40.0） | 14（28.0） |  |
| Delivery gestational week |  |  |  | 0.079 |
| Full term | 97（97.0） | 47（94.0） | 50（100.0） |  |
| Premature birth | 3（3.0） | 3（6.0） | 0（0） |  |
| Physical growth Z-score | |  |  |  |
| WAZ | 0.43（-0.28，1.05） | 0.57（-0.30，1.17） | 0.40（-0.31，1.04） | 0.741 |
| HAZ | 0.33（-0.24，0.88） | 0.31（-0.32，0.88） | 0.40（-0.12，0.92） | 0.448 |
| BAZ | 0.05（-0.47，0.80） | -0.14（-0.87，0.86） | 0.13（-0.36，0.76） | 0.237 |
| Nutritional status |  |  |  | 0.492 |
| Emaciation | 2（2.0） | 2（4.0） | 0（0） |  |
| Normal | 77（77.0） | 38（76.0） | 39（78.0） |  |
| Overweight | 17（17.0） | 8（16.0） | 9（18.0） |  |
| Obesity | 3（3.0） | 1（2.0） | 2（4.0） |  |
| Severe obesity | 1（1.0） | 1（2.0） | 0（0） |  |
| Back of appetite |  |  |  | 0.749 |
| Yes | 11（11.0） | 5（10.0） | 6（12.0） |  |
| No | 89（89.0） | 45（90.0） | 44（88.0） |  |
| Poor feeding interactions |  |  |  | 1 |
| Yes | 6（6.0） | 3（6.0） | 3（6.0） |  |
| No | 94（94.0） | 47（94.0） | 47（94.0） |  |

Categorical and continuous variables were shown as n (%) and median (p_25_, p_75_). BMI, Body mass index; HAZ, height for age Z-score; WAZ, weight for age Z-score; BAZ, BMI for age Z-score. **p*<0.05, ***p* <0.01.

**Supplementary Table2.** The comparisons of nutrient intake with EAR.

| Nutrients | ASD | TD | *p* |
| --- | --- | --- | --- |
|  | Percentage of children achieving EAR(%) | Percentage of children achieving EAR(%) |  |
| Ca | 50.0 | 84.0 | <0.001** |
| Male | 44.0 | 54.0 |  |
| Female | 6.0 | 30.0 |  |
| P | 98.0 | 96.0 | 0.558 |
| Male | 76.0 | 58.0 |  |
| Female | 22.0 | 38.0 |  |
| Zn | 92.0 | 92.0 | 1 |
| Male | 72.0 | 58.0 |  |
| Female | 20.0 | 34.0 |  |
| Vitamin A | 78.0 | 92.0 | 0.05 |
| Male | 60.0 | 56.0 |  |
| Female | 18.0 | 36.0 |  |
| Vitamin D | 30.0 | 80.0 | <0.001** |
| Male | 28.0 | 48.0 |  |
| Female | 2.0 | 32.0 |  |
| Vitamin C | 68.0 | 76.0 | 0.373 |
| Male | 56.0 | 52.0 |  |
| Female | 12.0 | 24.0 |  |
| Thiamine | 44.0 | 72.0 | 0.005* |
| Male | 38.0 | 46.0 |  |
| Female | 6.0 | 26.0 |  |
| Riboflavin | 60.0 | 88.0 | 0.001** |
| Male | 52.0 | 54.0 |  |
| Female | 8.0 | 34.0 |  |
| Vitamin B_12_ | 92.0 | 98.0 | 0.169 |
| Male | 72.0 | 60.0 |  |
| Female | 20.0 | 38.0 |  |

Ca, calcium; P, phosphorus; Zn, zinc.
